# MaxEnt Prediction of *Seriphidium transiliense* Habitats in China: Integrating Climate Change and Human Activity Effects

**DOI:** 10.1002/ece3.72641

**Published:** 2025-12-09

**Authors:** Wenxiong Li, Guili Jin, Yixuan Pan, WenXuan Zhao, Mengtian Chen, Chao Li, Wenlin Du

**Affiliations:** ^1^ Xinjiang Agriculture University Urumqi Xinjiang China

**Keywords:** climate change, human activities, MaxEnt model, *Seriphidium transiliense*, suitable habitat

## Abstract

*Seriphidium transiliense* is a key species in the arid and semi‐arid desert grasslands of Northwest China, playing crucial roles in maintaining ecological balance, stabilizing soil, and supporting biodiversity. However, the combined effects of global climate change and human activities are leading to a dramatic reduction in suitable habitat area, intensified habitat fragmentation, and ecosystem degradation. This study utilizes the MaxEnt model, along with field investigation data and online recorded data, selecting 159 effective occurrence points and integrating 20 environmental variables such as bioclimatic, soil, topographic, solar radiation, and human footprint factors, to evaluate the distribution patterns and dynamic changes of suitable habitats for *S. transiliense* under current climatic conditions and six future climate scenarios (SSP126, SSP245, SSP585). The results indicate that the total suitable habitat area for *S. transiliense* under current conditions is 86.20 × 10^4^ km^2^, mainly concentrated in northern Xinjiang. Human activities have drastically reduced the suitable habitat area, with the total area shrinking to 75.78 × 10^4^ km^2^, and the highly suitable habitat area decreasing from 5.72 × 10^4^ km^2^ to 2.00 × 10^4^ km^2^. Climate change in the future might expand its distribution range, but human activities continue to threaten its habitat, especially in areas of highly suitable habitat. The distribution center tends to migrate southeastward or northwestward under different climate scenarios, along with shifts in elevation. This research provides a scientific basis for the monitoring, protection, and ecological restoration of *S. transiliense* and underscores the necessity of scientific management and reseeding restoration amid escalating human activities.

## Graphical abstract

This study utilizes the MaxEnt model to evaluate the current and future potential distribution of *Seriphidium transiliense* in Northwest China, considering the impacts of climate change and human activities. The findings reveal that precipitation and human footprint are the most critical factors shaping the species' distribution, with suitable habitats expected to shift spatially under different climate scenarios. This research provides valuable insights for the ecological management and conservation of desert grasslands, contributing to the understanding of species distribution dynamics under dual environmental pressures.

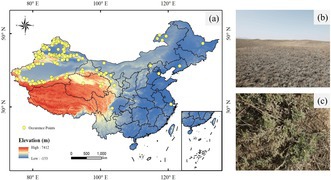

## 1 Introduction

*Seriphidium transiliense* serves as a foundational species in the desert grasslands of arid and semi‐arid regions in Northwest China and forms part of the larger Central Asian Desert Region. Renowned for its drought and salinity tolerance, it plays crucial ecological roles in maintaining balance within fragile ecosystems, stabilizing soil, and promoting biodiversity (Gao et al. 2016). Additionally, its well‐developed root system effectively prevents soil erosion, mitigates wind erosion, enhances soil fertility, and regulates regional microclimates (Jin et al. 2011). Despite its ecological importance, *S. transiliense* faces significant threats due to the intensification of global climate change and human activities such as overgrazing and land‐use changes (Amat et al. 2013). Nevertheless, the ecological consequences of these anthropogenic pressures on *S*. *transiliense* have not been systematically studied in Northwest China, and related research within the Central Asian Desert Region remains limited. These combined pressures from climate change and human activities have contributed to a long‐term reduction in its suitable habitats, exacerbating habitat fragmentation and accelerating ecosystem degradation.

Climate change directly affects the distribution range of *S. transiliense* by altering key environmental factors such as temperature and precipitation (Saymohammadi et al. 2017). Meanwhile, human activities indirectly disrupt ecological balance through changes in land use and overexploitation of resources, resulting in habitat fragmentation and biodiversity loss (Zhang et al. 2017). Climate change and human activities are the two core driving forces of current ecosystem changes: climate change directly alters the distribution patterns of species by modifying environmental conditions (García‐Valdés et al. 2018), while human activities exacerbate ecosystem degradation indirectly (Qing et al. 2024). For example, increased grazing intensity leads to the degradation and dwarfing of *S. transiliense* populations, while moderate grazing is beneficial for maintaining community structure stability (Kang et al. 2024). Furthermore, long‐term overgrazing and improper utilization have resulted in the degradation of *S. transiliense* desert grasslands, with some local areas experiencing extreme degradation or complete destruction.

To better assess the impact of climate change and human activities on the distribution of *S. transiliense*, species distribution models (SDMs) provide an effective tool (Zurell et al. 2020). By integrating known species distribution records with environmental variables, SDMs can predict current and potential distribution patterns and assess future trends. The Maximum Entropy model (MaxEnt) is widely used in ecological and conservation biology research due to its advantages in handling limited sample data and unbalanced distributions (Ahmadi et al. 2023). For instance, previous studies have utilized the MaxEnt model to analyze the potential suitable distribution areas of other plants and provide recommendations for optimizing protected areas (Ab Lah et al. 2021; Gao et al. 2022), demonstrating the efficiency and reliability of the MaxEnt model in predicting species distribution and analyzing ecological requirements.

Nevertheless, research on *S. transiliense* has mainly focused on localized areas in Xinjiang (Lu et al. 2009; Jin et al. 2011; Liang et al. 2013; Xia et al. 2015; Wu et al. 2019), with a lack of assessments at the national or broader spatial scale. Moreover, existing studies primarily focus on the singular impact of climate change on species distribution, while studies on the combined effects of climate change and human activities remain scarce. Therefore, systematically exploring the integrated effects of climate change and human activities on the distribution of *S. transiliense*, especially predicting its distribution dynamics under different future climate scenarios, has become a key research direction.

This study, based on an optimized MaxEnt model, combines multi‐source data such as bioclimatic, topographic, soil, solar radiation, and human footprint variables to systematically evaluate the driving mechanisms and future trends of *S. transiliense* distribution under the influence of climate change and human activities. The specific objectives include: (1) identifying key environmental factors limiting the distribution of *S. transiliense* and quantifying the relative contributions of climate change and human activities to its distribution patterns; (2) predicting the spatiotemporal dynamic changes of suitable habitats under different future climate scenarios (2020–2060); (3) analyzing potential migration paths of the distribution center and proposing scientific and rational management recommendations. The results of this study will provide a deeper understanding of the distribution characteristics and future trends of *S. transiliense*, offering theoretical support and decision‐making guidance for formulating scientifically sound ecological restoration strategies.

## 2 Materials and Methods

### 2.1 Species Occurrence Data

**FIGURE 1:**
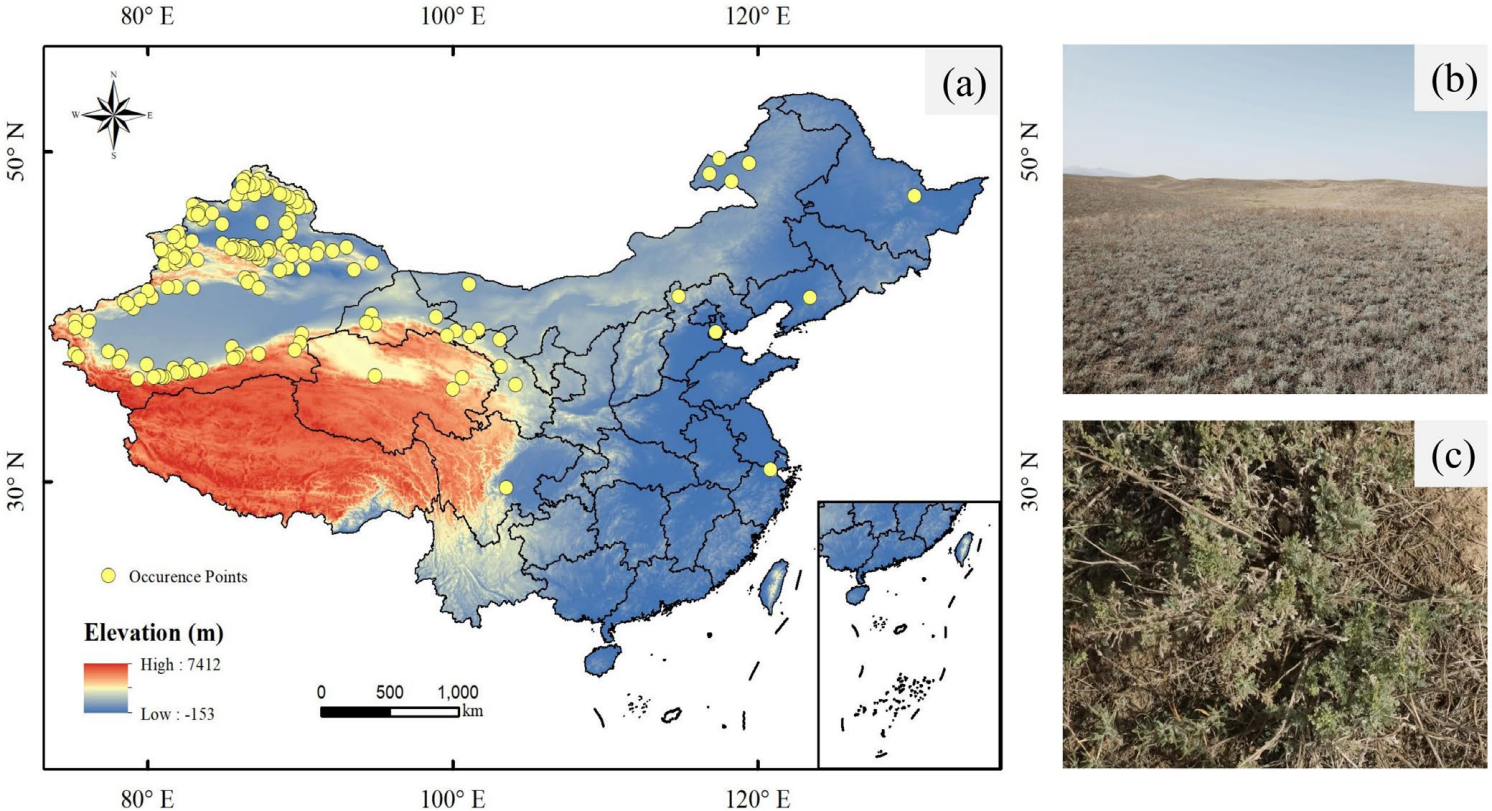
(a) Occurrence Records of *Seriphidium transiliense* Populations in China, (b) Landscape of *S. transiliense*, (c) Single *S. transiliense*.

Species occurrence data for *S. transiliense* were collected using three approaches:(1) Field surveys conducted by our research team in Xinjiang during 2021–2022, during which GPS devices were used to record the latitude, longitude, and elevation of individual *S. transiliense* plants. These surveys provided primary occurrence data for this study.(2) Data retrieved from the Global Biodiversity Information Facility (GBIF, https://www.gbif.org/). (3) Data obtained from the Chinese Virtual Herbarium (http://www.cvh.ac.cn/). These three sources yielded 68, 571, and 34 occurrence records for *S. transiliense* in China, respectively. To avoid spatial autocorrelation between occurrence points, duplicate points within a distance of less than 10 km were removed. A 10 km × 10 km grid was created in ArcGIS 10.8, and only one distribution point was retained within each grid whenever possible. Ultimately, 159 valid occurrence points were identified (Figure 1). The administrative map of China used in this study was sourced from the National Geospatial Information Resource Catalog Service System (https://www.webmap.cn/).

### 2.2 Environmental Variables

In this study, 39 environmental variables potentially influencing the distribution of *Seriphidium* species were initially selected, including bioclimatic, soil, topographic, solar radiation, and human footprint data. All selected variables were resampled using bilinear interpolation, and the spatial resolution of all variables was unified to 2.5 km. The bioclimatic data were derived from the WorldClim dataset version (https://worldclim.org/), which consists of precipitation and temperature variability (Eyring et al. 2016). Elevation data were obtained from the WorldClim dataset, while slope and aspect data were extracted from elevation data using ArcGIS software. Current solar radiation data were also sourced from the WorldClim dataset, while future solar radiation data were acquired from CMIP6 (https://esgf‐node.llnl.gov/search/cmip6/). Soil data were downloaded from the Harmonized World Soil Database version 1.2 (HWSD). Human footprint (Hf) data were sourced from the Global Human Interference Dataset version 3, which objectively reflects the intensity and spatial distribution of human activities. These data were processed using weighted standardization based on land use, population density, transportation networks, cropland, and grazing data from 1993 to 2009, representing the degree of human disturbance (Venter et al. 2016). In this study, the Hf variable serves as a proxy for anthropogenic pressure on the sensitive habitats of *Seriphidium* species. A higher Hf value is interpreted as reflecting greater ecological stress from factors such as grazing and land conversion, thereby acting as a limiting factor on the species' distribution within the model. This increased human pressure limits the potential distribution of Seriphidium species by reducing suitable habitats and intensifying competition for resources. Therefore, in our model, areas with high Hf values are considered less suitable for the species' survival and distribution, reflecting the impact of human activities on ecological integrity.

This study used current bioclimatic data from 1970 to 2000 and future bioclimatic data from 2020 to 2040 and 2040 to 2060. Future climate data were based on the BCC‐CMS2‐MR Global Climate Model (GCM) in the IPCC Sixth Assessment Report, with three Shared Socioeconomic Pathways (SSPs) selected: SSP126 (low‐carbon pathway, representing an environmentally friendly future that mitigates climate change impacts), SSP245 (medium‐carbon pathway, reflecting a continuation of current policy trends), and SSP585 (high‐carbon pathway, resulting in severe climate change and socioeconomic risks) (Eyring et al. 2016). By combining the two time periods (2020–2040 and 2040–2060) with the three pathways, six future climate scenarios were formed: SSP126–2040, SSP126–2060, SSP245–2040, SSP245–2060, SSP585–2040, and SSP585–2060.

To avoid issues of autocorrelation and multicollinearity among variables (Worthington et al. 2016), Spearman's correlation coefficient was used to screen the variables. Variables with correlation coefficients < |0.70| were selected for modeling. The jackknife method was applied to determine the contribution of each variable to model predictions. Variables with low contribution rates were excluded. Based on species ecology, existing literature, expert judgment, and correlation tests, a final set of 20 variables was selected for modeling (Table 1).

**TABLE 1:** Variable name and unit.

Variables	Description	Unit
bio4	Temperature seasonality	Standard deviation ×100
bio5	Max temperature of warmest month	°C
bio7	Temperature annual range (bio5‐bio6)	%
bio9	Mean temperature of driest quarter	°C
bio11	Mean temperature of coldest quarter	°C
bio13	Precipitation of wettest month	mm
bio15	Precipitation seasonality	Coefficient of variation
bio16	Precipitation of wettest quarter	mm
altitude	Altitude	m
aspect	Aspect	°
slope	Slope	°
t_bs	Topsoil BaseSaturation	%
t_caco3	Topsoil calcium carbonate	%weight
t_caso4	Topsoil gypsum	%weight
t_cec_clay	Topsoil CEC (clay)	cmol/kg
t_ece_soil	Topsoil CEC (soil)	cmol/kg
t_gravel	Topsoil gravel content	%vol
t_silt	Topsoil silt fraction	%wt
solar radiation	Solar radiation	KJ/m^2^/day
Hf	Human footprint	—

### 2.3 Construction and Optimization of the MaxEnt Model

Three types of models were constructed to investigate the impact of environmental changes and human activities on the distribution patterns of *S. transiliense*: *Model H1*: Predictions based on environmental variables (bioclimatic, topographic, soil, and solar radiation) under the current climate scenario. Model H2: Predictions based on environmental variables (bioclimatic, topographic, soil, and solar radiation) plus human footprint under the current climate scenario. Model F: Predictions based on environmental variables (future bioclimatic, soil, topographic, and future solar radiation) under future climate scenarios. Models H1 and H2 were both based on current climate scenarios.

The MaxEnt SDM, an ecological niche model, is based on the principle of maximum entropy and uses species presence data along with environmental variables to predict the potential distribution of species (Hosseini et al. 2024). This study employed MaxEnt version 3.4.4, running in a JAVA 21 environment (https://www.oracle.com). The Enmeval package was used to optimize the model by tuning two parameters: the Regularization Multiplier (RM) and Feature Combination (FC) (Kass et al. 2021). During optimization, RM values ranged from 0.5 to 6, increasing by 0.5 increments to provide 12 regularization multipliers. FC included six combinations: H, L, LQ, LQH, LQHP, and LQHPTL.

The difference between the AUC values of the training and test datasets (avg.AUCDIFF) was used to assess the consistency of the model's performance across these datasets. A smaller difference indicates a stronger generalization ability of the model. The study applied the corrected Akaike Information Criterion (AICc) for model selection. AICc improves upon the Akaike Information Criterion (AIC) by accounting for both model fit and complexity, aiming to find a model that fits the data well without being overly complex. Under the AICc criterion, lower delta.AICc (Delta Akaike Information Criterion corrected) scores indicate better models (Ogasawara 2016).

### 2.4 Evaluation of the MaxEnt Model

To evaluate the accuracy of the modeling results, the Area Under the Receiver Operating Characteristic (ROC) Curve (AUC) was calculated. AUC is a powerful tool for assessing model performance and is independent of threshold selection (Wan et al. 2019). The AUC indicates the model's ability to distinguish between presence and random background. AUC values range from 0 to 1.0, where 0.5 represents random prediction performance, and 1.0 indicates perfect discrimination.

The Percent Contribution (PC) from the Jackknife test was used to evaluate the relative importance of each environmental variable influencing the species model under current climate conditions. This is an important metric in studies with small sample sizes (Wu et al. 2024) and helps identify the relative significance of different environmental factors in shaping the species' habitat. Additionally, the True Skill Statistic (TSS = Sensitivity + Specificity—1) is a straightforward and intuitive metric used to measure the predictive performance of SDMs (Allouche et al. 2006). TSS ranges from −1 to 1, with the following interpretations: poor (−1 to 0.4), fair (0.4–0.5), good (0.5–0.7), very good (0.7–0.85), excellent (0.85–0.9), and nearly perfect to perfect (0.9–1) (Gebrewahid et al. 2024; Xu et al. 2024).

In this study, AUC and TSS were combined to comprehensively demonstrate the predictive performance of the MaxEnt model (Leroy et al. 2018). While AUC reflects the model's overall ability to distinguish between positive and negative cases, TSS emphasizes the classification performance under specific thresholds, making it particularly advantageous for handling imbalanced data. The combination of these two metrics provides a more comprehensive and accurate evaluation of the model's reliability and applicability.

### 2.5 Prediction and Zoning of Suitable Habitats

After running the MaxEnt model simulations, ArcGIS software was used to convert the ASCII files containing the average results of 10 repetitions for each period into Raster format. The raster data represent the survival probability of *S. transiliense*. The potential suitable distribution areas of Seriphidium species were then divided into four categories using the Jenks method, combined with the species' actual distribution patterns and predicted probability values (*p*): Non‐suitable areas (0 ≤ *p* < 0.2), Low‐suitability areas (0.2 ≤ *p* < 0.5), Medium‐suitability areas (0.5 ≤ *p* < 0.75), and High‐suitability areas (0.75 ≤ *p* ≤ 1). Finally, through reclassification, raster calculations, and zonal statistics, the area changes of suitable habitats were calculated for each time period (Jia et al. 2024; Xie et al. 2024).

## 3 Results and Analysis

### 3.1 Evaluation of Model Prediction Results

The MaxEnt model was optimized using the ENMeval package in R Studio. The optimization process determined the best parameter settings (Table 2): Feature Classes (FC) as LQ, Regularization Multiplier (RM) as 1.5, and a minimum difference in the Akaike Information Criterion corrected (delta.AICc = 0). The optimized parameters significantly reduced the degree of model overfitting while improving the prediction accuracy of the model.

**TABLE 2:** Evaluation index based on Enmeval optimization.

Type	FC	RM	delta.AICc	avg.diff.AUC
Default	LQHPT	1.00	116.79	0.058
Optimized	LQ	1.50	0.00	0.027

The optimization results indicated that, compared to the default settings, the adjusted model reduced the AICc value and exhibited a lower AUC difference, thereby enhancing the overall performance and applicability of the model.

In this study, we used AUC and TSS values to jointly evaluate the simulation prediction results (Table 3). Under the MaxEnt model, the average training AUC value was 0.935, the average test AUC value was 0.915, and the average TSS value was 0.850, indicating good accuracy of the model's predictions. When the Regularization Multiplier (RM) was set to 1.5 and Feature Classes (FC) to LQ, the MaxEnt model performed best, with a training AUC value of 0.968, a test AUC value of 0.927, and a TSS value of 0.895. Moreover, a comparison across different climate scenarios showed that the model maintained strong predictive robustness, with testing AUC values ranging from 0.903 to 0.920 and TSS values from 0.823 to 0.840. These results suggest stable and reliable model performance under various future climate conditions.

**TABLE 3:** Model accuracy evaluation.

Model	Scenario	Training AUC	Testing AUC	TSS
H1	Current	0.968	0.927	0.895
H2	Current	0.971	0.932	0.903
F1	SSP126–2040	0.927	0.907	0.834
F2	SSP126–2060	0.915	0.908	0.823
F3	SSP245–2040	0.926	0.903	0.830
F4	SSP245–2060	0.928	0.910	0.839
F5	SSP585–2040	0.920	0.920	0.840
F6	SSP585–2060	0.927	0.911	0.838

**FIGURE 2:**
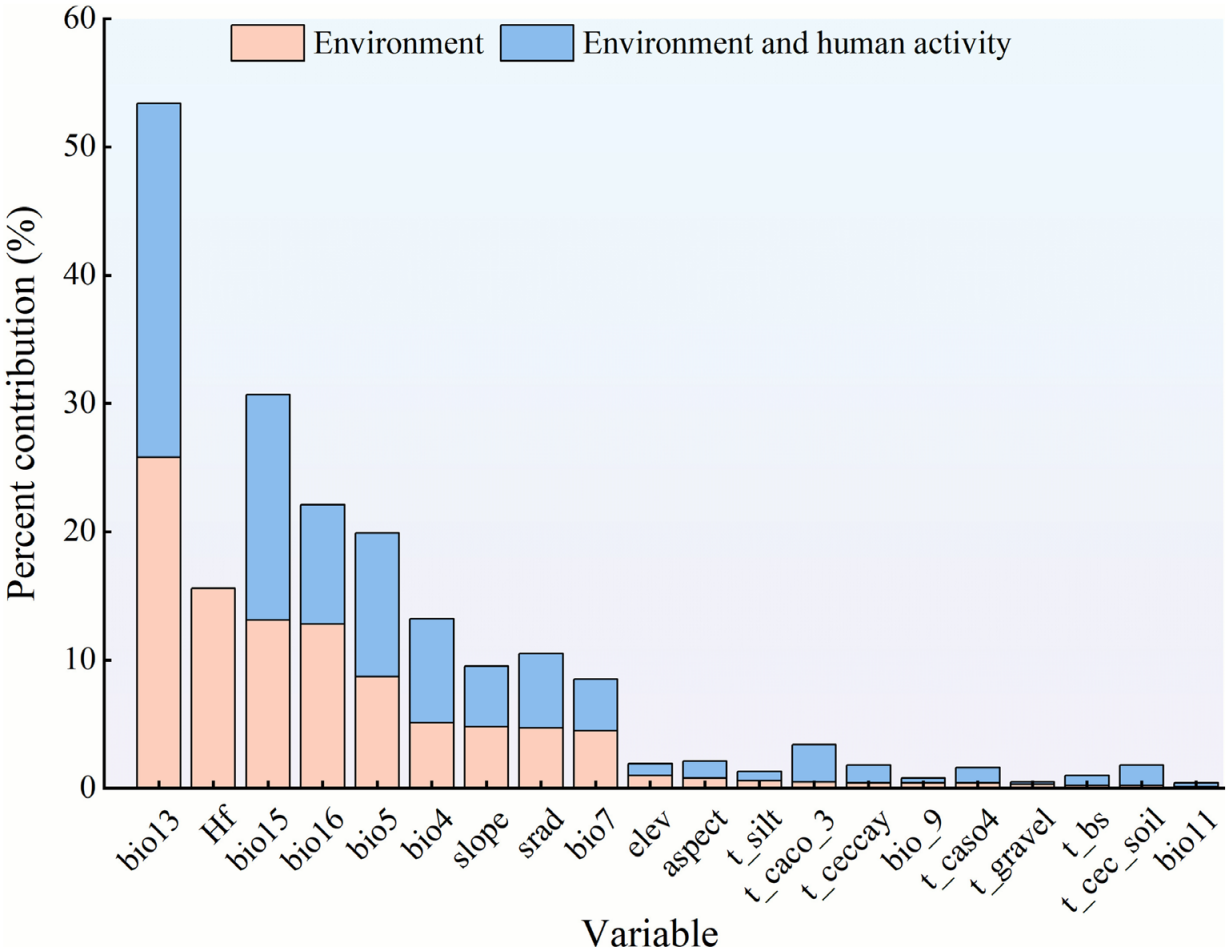
Contribution Rates of Environmental Variables in the MaxEnt Model:Blue (Environment): Represents the contribution rates of environmental variables only (from Model H1), and Orange (Environment and Human Activity): Represents the contribution rates of environmental variables combined with human activities (from Model H2).

It is generally considered that when the cumulative contribution rate of variables exceeds 85%, these variables are regarded as the primary factors (Yang et al. 2022). The MaxEnt model prediction results (Figure 2) indicate that under environmental influences (Model H1), the primary variables affecting the potential geographic distribution of *S. transiliense* are bio13 (27.6%), bio15 (17.6%), bio5 (11.2%), bio16 (9.3%), bio4 (8.1%), solar radiation (5.8%), slope (4.7%), and bio7 (4.0%), with a cumulative contribution rate of 88.3%. With the inclusion of human activity disturbances (Model H2), the primary variables affecting the potential geographic distribution of *S. transiliense* are bio13 (25.8%), Hf (15.6%), bio15 (13.1%), bio16 (12.8%), bio5 (8.7%), bio4 (5.1%), and slope (4.8%).

### 3.2 Prediction of *S. transiliense* Habitats Under Current Climate and Human Activity Disturbance

**FIGURE 3:**
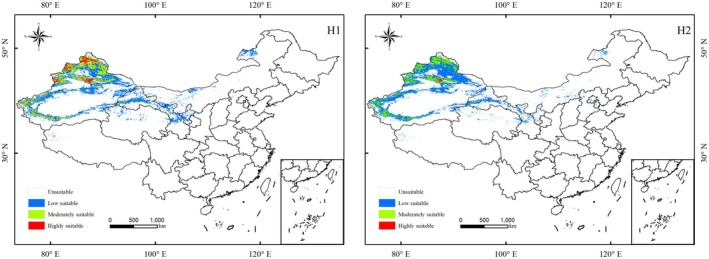
Suitable habitat distribution of *Seriphidium transiliense* under current climate scenarios without (H1) and with (H2) human activity disturbance.

The distribution area of *S. transiliense* with and without human activity disturbances was predicted using the MaxEnt model (Figure 3), and the habitat suitability was classified into levels to calculate the suitable habitat areas for each category (Table 4). Under the current climate scenario, the total suitable habitat area of *S. transiliense* in China, influenced by environmental factors alone, is 86.20 × 10^4^ km^2^. These habitats are primarily distributed across the Xinjiang Uygur Autonomous Region, Inner Mongolia Autonomous Region, Gansu Province, Qinghai Province, Ningxia Hui Autonomous Region, Tibet Autonomous Region, Shaanxi Province, and Hebei Province (Figure 3, H1). The area of highly suitable habitat is 5.72 × 10^4^ km^2^, primarily distributed in northern Xinjiang. The moderately suitable habitat area is 23.02 × 10^4^ km^2^, mainly located in northern Xinjiang, northern Gansu, and western Inner Mongolia. The low‐suitability habitat area is 57.46 × 10^4^ km^2^, primarily distributed in northern Xinjiang, central and southern Gansu, northern Qinghai, and western Ningxia.

**TABLE 4:** Suitable habitat area of *S. transiliense* with and without human activity disturbance (×10^4^ km^2^).

Human activity	Highly suitable habitat	Moderately suitable habitat	Low suitable habitat	Unsuitable habitat
Without human activity	5.72	23.02	57.46	873.49
With human activity	2.00	17.84	55.94	883.92

With the inclusion of human activity disturbance, the total habitat area of *S. transiliense* in China is reduced to 75.78 × 10^4^ km^2^. Compared to the scenario influenced only by environmental factors, the overall distribution pattern of *S. transiliense* habitats remains similar, but the total suitable habitat area decreases by 12.09%. The areas of suitable habitats at all levels are reduced: the highly suitable habitat area decreases by 1.52 × 10^4^ km^2^, and the moderately suitable habitat area decreases by 5.18 × 10^4^ km^2^ (Figure 3, H2).

### 3.3 Changes in the Spatial Distribution Pattern of *S. transiliense* Under Different Climate Change Scenarios

**FIGURE 4:**
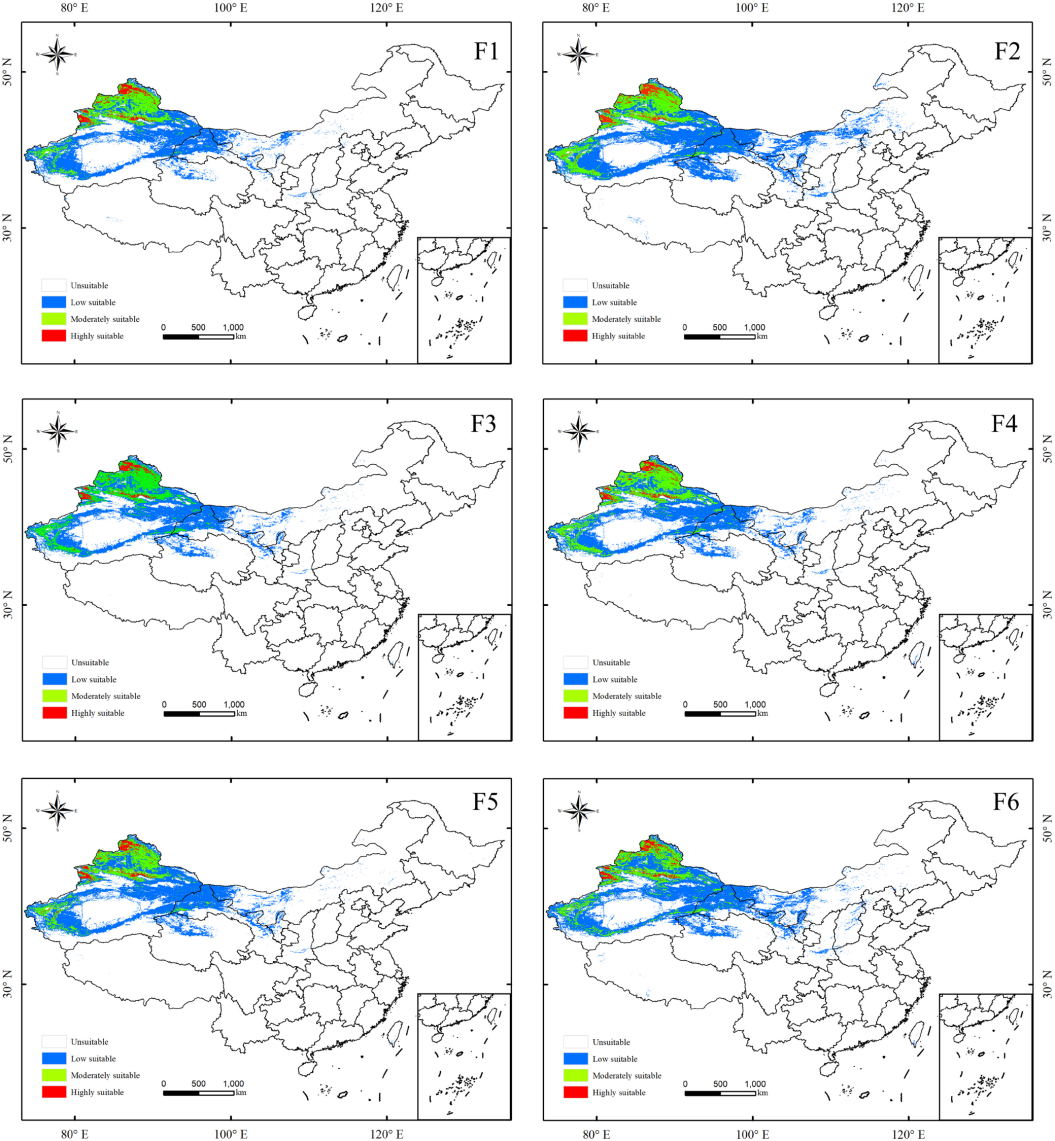
Predicted changes in the potential habitat of *Seriphidium transiliense* Under SSP126 (F1, F2), SSP245 (F3, F4), and SSP585 (F5, F6) Scenarios for 2020–2040 (F1, F3, F5) and 2040–2060 (F2, F4, F6).

Under future climate scenarios, the predicted suitable habitat range of *S. transiliense* is generally consistent with that under current climate conditions. The habitats are mainly distributed in the Xinjiang Uygur Autonomous Region, Inner Mongolia Autonomous Region, Gansu Province, Qinghai Province, Ningxia Hui Autonomous Region, and Tibet Autonomous Region. Under different future climate scenarios, the highly and moderately suitable habitats are primarily located in northern and western Xinjiang, while the low‐suitability habitats are mainly distributed in eastern Xinjiang, northern Gansu, northern Qinghai, western Inner Mongolia, and central Shaanxi (Figure 4).

**FIGURE 5:**
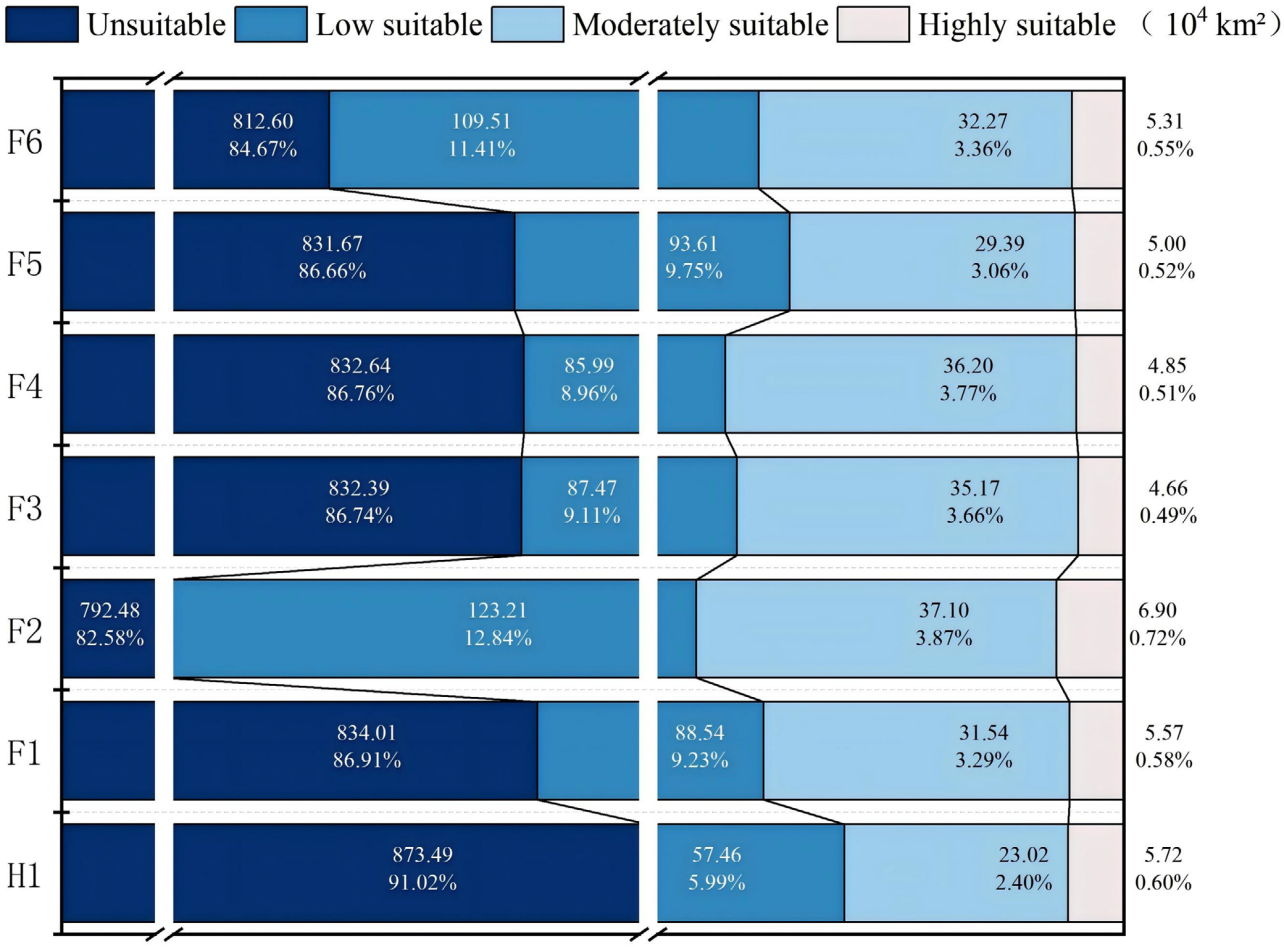
Suitable habitat and proportion of *Seriphidium transiliense* under different climate change scenarios.

The differences between the predicted suitable habitat area of *S. transiliense* under future climate scenarios and that under current climate scenarios are relatively small. Under the current climate scenario, the total suitable habitat area of *S. transiliense* is 86.20 × 10^4^ km^2^, accounting for 8.98% of the total land area. Under future climate scenarios, highly suitable habitats are predicted to remain primarily in northern Xinjiang, with an area ranging from 4.66 × 10^4^ km^2^ to 6.90 × 10^4^ km^2^, corresponding to a percentage range of 0.49%–0.72%, showing only minor differences from the highly suitable habitat area under the current climate scenario. Among the future climate models, Model F2 (SSP126‐2060) predicts the largest suitable habitat area for *S. transiliense*, while Model F3 (SSP245‐2040) predicts the smallest suitable habitat area (Figure 5).

Area percentage analysis shows that over time (2000, 2040, 2060), the changes in the highly suitable habitat area of *S. transiliense* under the SSP126, SSP245, and SSP585 climate scenarios exhibit consistent trends. Specifically, the change rate of highly suitable habitat area shows a trend of first decreasing and then increasing, with an overall change rate of 0.03%–0.12%.

This indicates that under varying CO_2_ emission levels, the differences in *S. transiliense* habitat area changes tend to stabilize over time. Climate change will lead to an increase in moderately suitable habitat areas for *S. transiliense* in northern Xinjiang, while low‐suitability habitat areas are expected to decrease in central Shaanxi, southern Gansu, northern Ningxia, and western Inner Mongolia. However, low‐suitability habitat areas are predicted to increase in eastern Taiwan (Figure 4).

Among the models, the largest difference is observed between Model F2 (SSP126–2060) and Model H (current), particularly in the low‐suitability habitat areas in southern and northern Xinjiang, central and northern Gansu, central Shaanxi, northern Ningxia, and western Inner Mongolia, with an increase of 65.75 × 10^4^ km^2^ (Figure 5).

### 3.4 Spatial Variation of *S. transiliense* and Shifts in Its Potential Distribution Center

The “Overlap Analysis” tool in ArcGIS was used to analyze the distribution results of *S. transiliense* in different time periods under the same shared socioeconomic pathway (SSP) (Figure 4). The results show that for the three different SSPs, the changes in expansion area, reduction area, and unchanged area of *S. transiliense* follow the pattern: low suitability > moderate suitability > high suitability, and unchanged area > reduction area > expansion area.

Additionally, the expansion areas for high, medium, and low suitability habitats all exhibited only slight changes (0–0.13 × 10^4^ km^2^), while the reduction areas for medium and low suitability habitats showed substantial variations (17.51–84.19 × 10^4^ km^2^, Table 5). The expansion and contraction of low‐suitability habitats for *S. transiliense* mainly occurred in northern Xinjiang and northern Gansu, while the areas of moderate‐ and high‐suitability habitats in northern Xinjiang remained largely unchanged. Moreover, the areas of suitable habitats at different levels for *S. transiliense* varied with changes in CO^2^ concentration (Figure 5).

**TABLE 5:** Spatial changes of *Seriphidium transiliense* under different climate conditions (×10^4^ km^2^).

Model	Highly suitable habitat			Moderately suitable habitat			Low suitable habitat		
	Gain	Loss	Unchanged	Gain	Loss	Unchanged	Gain	Loss	Unchanged
F1	0.00	3.12	2.44	0.02	17.65	13.81	0.06	49.55	38.78
F2	0.00	4.71	2.17	0.01	25.35	11.66	0.04	84.19	38.73
F3	0.00	2.54	2.11	0.02	19.17	15.90	0.06	47.67	39.56
F4	0.01	2.73	2.09	0.05	20.38	15.64	0.13	48.42	37.14
F5	0.00	3.00	1.98	0.02	17.63	11.64	0.05	56.15	37.08
F6	0.00	2.88	2.41	0.02	17.51	14.66	0.07	59.43	49.74

Under the F4 model (SSP245–2060), *S. transiliense* shows the largest expansion area of 0.19 × 10^4^ km^2^, mainly distributed in northern Xinjiang, including Urumqi, Changji Prefecture, and Altay Prefecture. The smallest expansion area occurs under the F2 model (SSP126–2060), with 0.05 × 10^4^ km^2^, primarily located in northern Xinjiang, including Tacheng Prefecture and Altay Prefecture.

Under the F2 model (SSP126–2060), *S. transiliense* shows the largest contraction area of 114.25 × 10^4^ km^2^, mainly distributed in northern Xinjiang, Kashgar, and western Hotan regions. The smallest contraction area occurs under the F3 model (SSP245–2040), with 69.38 × 10^4^ km^2^, primarily distributed in northern Xinjiang, Kashgar, and western Hotan regions.

Under the F6 model (SSP585–2060), *S. transiliense* shows the largest unchanged area of 66.81 × 10^4^ km^2^, mainly located in eastern and western Xinjiang, and central and western Inner Mongolia. The smallest unchanged area occurs under the F2 model (SSP126–2060), with 52.56 × 10^4^ km^2^, primarily in eastern and western Xinjiang.

By utilizing the “Centroid Shift Analysis (Linear)” module in SDMtoolbox, simulations were conducted for three different SSPs to evaluate changes in the centroid position of suitable habitats for the 2040s and 2060s, comparing them with the current centroid position to explore overall trends in habitat distribution changes.

**FIGURE 6:**
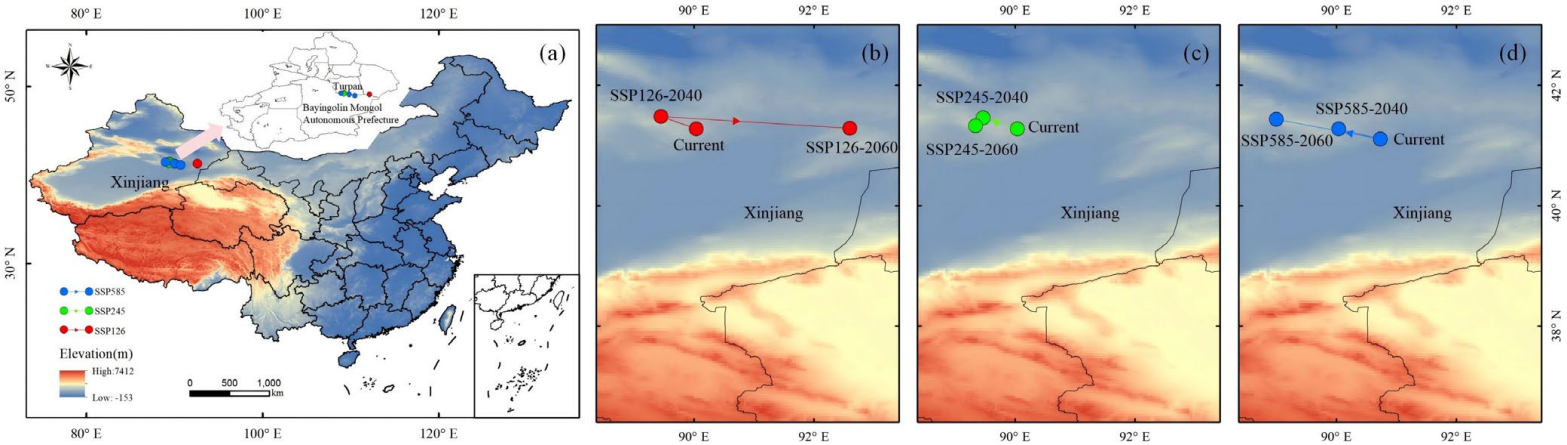
(a) Potential distribution centers of *Seriphidium transiliense* Under SSP126 (b), SSP245 (c), and SSP585 (d).

The results indicate that the current distribution center of *S. transiliense* is located in southern Turpan, Xinjiang. Under the SSP126 scenario, the distribution center exhibits a southeastward migration trend in the horizontal gradient. In contrast, under the SSP245 and SSP585 scenarios, the distribution center shows a northwestward migration trend in the horizontal gradient. In the vertical gradient, the SSP126 and SSP585 scenarios demonstrate a tendency for the distribution center to shift to lower altitudes, while the SSP245 scenario shows a shift to higher altitudes (Figure 6).

Under the current climate scenario, the distribution center of *S. transiliense* is located in northern Bayingolin Mongol Autonomous Prefecture, Xinjiang, at coordinates 41.28°N, 90.03°E, with an elevation of 1451 m. Under SSP126, the distribution center for the 2040s is at 41.48°N, 89.46°E, with an elevation of 1328 m, and for the 2060s at 41.28°N, 92.62°E, with an elevation of 1290 m. The horizontal centroid shifted 214.68 km eastward, and the vertical centroid moved 161 m to lower elevations (Figure 6b). Under SSP245, the distribution center for the 2040s is at 44.20°N, 84.31°E, with an elevation of 1484 m, and for the 2060s at 41.33°N, 89.35°E, with an elevation of 1535 m. The horizontal centroid shifted 59.24 km northwestward, and the vertical centroid moved 84 m to higher elevations (Figure 6c). Under SSP585, the distribution center for the 2040s is at 41.10°N, 90.75°E, with an elevation of 886 m, and for the 2060s at 41.43°N, 89.02°E, with an elevation of 1496 m. The horizontal centroid shifted 90.25 km northwestward, and the vertical centroid moved 51 m to lower elevations (Figure 6d).

## 4 Discussion

### 4.1 Analysis of Key Environmental Variables

According to the MaxEnt model predictions, the most important variables influencing the potential geographic distribution of *S. transiliense* under current climate change and human disturbance conditions are precipitation of the wettest month (bio13), human footprint (Hf), precipitation seasonality (bio15), and precipitation of the wettest quarter (bio16). The optimal growth conditions for *S. transiliense* are: bio13 ranging from 3 to 66 mm, Hf from 0 to 46, bio15 from 12.49 to 22.66 mm, and bio16 from 8 to 186 mm.

Precipitation is a critical factor influencing plant growth and spatial distribution (Holdrege et al. 2021; Lu et al. 2023). In arid and semi‐arid regions, water availability is the primary limiting factor. As a desert plant, *S. transiliense* shows strong adaptability to changes in precipitation, especially during the wettest periods, when sufficient precipitation is crucial for replenishing soil moisture and maintaining plant metabolic activities. The significant contributions of bio13 and bio16 to the model indicate that *S. transiliense* distribution is highly dependent on water availability during the growing season (He et al. 2023). Meanwhile, bio15 reflects the plant's sensitivity to temporal fluctuations in water availability, as irregular precipitation patterns can significantly impact germination and survival (Augspurger 1979).

In addition to climatic factors, human footprint (Hf) was identified as a key variable, contributing 15.60% to the model. This highlights the significant impact of human activities on *S. transiliense* habitats. The inclusion of Hf reduced the total suitable habitat area by 12.09%, with significant reductions in both highly and moderately suitable habitat areas. Human activities often lead to habitat fragmentation, soil degradation, and increased competition from invasive species (Laurance et al. 2009; Wang et al. 2024), all of which pose threats to desert plants. The high contribution rate of Hf underscores the importance of incorporating anthropogenic factors into conservation planning for this species.

### 4.2 Geographic Distribution Characteristics of Suitable Habitats

Global warming has caused changes in temperature and precipitation patterns, which can lead to plant migration and shifts in distribution patterns when climatic conditions approach or exceed their adaptive thresholds (Parmesan and Yohe 2003). Under future climate change scenarios, the overall pattern of suitable habitats for *S. transiliense* remains relatively stable. The change in the area of highly suitable habitats is minimal, indicating the plant's certain tolerance and adaptability to environmental changes (Hochfeld and Hinners 2024).

However, the specific distribution centers and areas of suitable habitats show dynamic changes under different scenarios (Wang and Wu 2025), revealing complex ecological response patterns (Zhang et al. 2024). Under the SSP126 scenario, the distribution center shifts southeastward, while under SSP245 and SSP585 scenarios, it migrates northwestward. Vertically, the distribution center moves to lower altitudes under SSP126 and SSP585 scenarios, but shifts to higher altitudes under SSP245, possibly due to interactions between precipitation and temperature under different scenarios (Lu et al. 2020). These shifts are closely related to regional differences in precipitation and temperature (He et al. 2021), reflecting *S. transiliense*'s sensitivity to water availability and climatic suitability, as well as the relative climatic stability of northern Xinjiang in supporting its distribution.

The dynamic changes in highly suitable habitats exhibit a “decrease‐then‐increase” trend, which may be attributed to initial fluctuations in the spatial and temporal distribution of precipitation limiting germination and growth windows (Hu et al. 2018), followed by the gradual stabilization of precipitation patterns mitigating these effects (Ru et al. 2024). Additionally, the model predicts slight expansions of low‐suitability habitats (e.g., in eastern Xinjiang and southern Gansu) under future scenarios, which may be associated with improved regional precipitation conditions or rising temperatures, offering new possibilities for potential ecological restoration.

The predicted shifts in the distribution center of *S. transiliense* could have significant ecological implications. As the species migrates under different climate change scenarios, it may encounter new competitive pressures from other desert grassland species, which could affect its establishment, survival, and reproduction. Additionally, changes in the species' range may alter its interactions with pollinators and herbivores, potentially affecting mutualistic relationships. These shifts may also have cascading effects on ecosystem services, such as soil stabilization and biodiversity support. Given *S. transiliense*'s role as a foundational species, its distribution changes could impact the overall structure and functioning of desert grassland ecosystems, influencing other species that rely on the same ecological functions (Zhang et al. 2024).

### 4.3 Recommendations for Ecological Restoration of *S*. *Transiliense* Grasslands

Based on the MaxEnt model predictions, the suitable habitats for *S. transiliense* are primarily concentrated in northern Xinjiang. However, under the dual pressures of climate change and human activities, its suitable habitat area, particularly the highly suitable habitats, has significantly decreased. This highlights the need to focus future conservation efforts on monitoring and managing these core areas to reduce human activity and promote ecological restoration (Luo et al. 2024). The study reveals that the distribution centroids shift under different climate scenarios, reflecting the importance of climatic factors in driving species distribution while identifying some potential expansion habitats. These areas should be included in long‐term monitoring and evaluation (Kelly and Goulden 2008; Zu et al. 2023).

To achieve sustainable protection, especially in the northern part of Xinjiang, grassland degradation should be reduced through scientific grazing strategies, and ecological compensation or grazing ban policies should be introduced (Chen and Chen 2024). Based on the species' precipitation requirements, reseeding protocols should be developed and applied in degraded areas to enhance habitat recovery, by considering the species' optimal precipitation range to increase the likelihood of successful establishment and growth (Gao et al. 2016). Additionally, raising public awareness of the importance of desert plant conservation is essential. These integrated measures will provide crucial support for mitigating the pressures of climate change and human activities, maintaining regional ecosystem functions, and ensuring the long‐term stability of *S. transiliense* habitats.

### 4.4 Limitations

This study has certain limitations. For example, species distribution data were primarily derived from existing databases and field surveys, which may not fully reflect the actual distribution range of *S. transiliense*. Additionally, while the independent and combined effects of climatic factors and human activities were preliminarily evaluated, the complex interactions between the two were not explored in depth. Future studies could incorporate ecological process models or integrated dynamic models (Wang et al. 2020, 2021) to comprehensively assess the long‐term impacts of climate change and human activities on desert plants. Furthermore, conducting multi‐model comparison analyses using more global climate models (GCMs) could improve the robustness and reliability of the predictions (Guan et al. 2021; Chen et al. 2024), providing more scientific evidence for the conservation and management of desert plants.

## 5 Conclusion

In this study, a SDM was constructed using the MaxEnt model, integrating bioclimatic, soil, topographic, solar radiation, and human footprint data to investigate the impacts of climate change and human activities on the geographic distribution of *S. transiliense*. The results indicate that human activities significantly inhibit the spread of suitable habitats for *S. transiliense*, while climate change promotes the expansion of its distribution, primarily in northern Xinjiang and adjacent regions. With rising global temperatures and precipitation, *S. transiliense* is likely to migrate northwestward along the horizontal gradient and to higher altitudes along the vertical gradient in response to ecological pressures caused by human activities and climate change. These findings provide scientific support for the monitoring, conservation, and management of *S. transiliense*, while also offering important insights into the long‐term impacts of climate change and human activities on desert plants.

## Author Contributions

**Wenxiong Li:** conceptualization (lead), data curation (lead), formal analysis (lead), investigation (lead), methodology (lead), writing – original draft (lead), writing – review and editing (lead). **Guili Jin:** conceptualization (lead), funding acquisition (supporting), methodology (lead), supervision (lead), writing – original draft (lead), writing – review and editing (lead). **Yixuan Pan:** conceptualization (equal), data curation (equal), formal analysis (lead), investigation (lead), methodology (lead), writing – original draft (equal), writing – review and editing (equal). **WenXuan Zhao:** conceptualization (equal), resources (equal). **Mengtian Chen:** formal analysis (equal), resources (equal). **Chao Li:** investigation (equal), resources (equal). **Wenlin Du:** investigation (equal), resources (equal).

## Funding

This work was supported by the National Natural Science Foundation of China (31960360).

## Conflicts of Interest

The authors declare no conflicts of interest.

## Data Availability

All data used in this study are included in the paper and are available.
